# Application of a targeted amplicon sequencing panel to screen for insecticide resistance mutations in *Anopheles darlingi* populations from Brazil

**DOI:** 10.1038/s41598-024-84432-x

**Published:** 2025-01-03

**Authors:** Holly Acford-Palmer, Alice O. Andrade, Jody E. Phelan, Rosa A. Santana, Stefanie C. P. Lopes, Jansen F. Medeiros, Taane G. Clark, Maisa S. Araujo, Susana Campino

**Affiliations:** 1 Faculty of Infectious and Tropical Diseases, London School of Hygiene and Tropical Medicine, London, UK; 2 Plataforma de Produção e Infecção de Vetores da Malária- PIVEM, Laboratório de Entomologia, Fiocruz Rondonia, Porto Velho, RO Brazil; 3 Instituto de Pesquisa Clínica Carlos Borborema, Fundação de Medicina Tropical Dr. Heitor Vieira Dourado, Manaus, AM Brazil; 4 Instituto Leônidas & Maria Deane, FIOCRUZ, Manaus, AM Brazil; 5 Faculty of Epidemiology and Population Health, London School of Hygiene and Tropical Medicine, London, UK; 6 Programa de Pós-Graduação em Biologia Experimental – PGBIOEXP, Fundação Universidade Federal de Rondonia, Porto Velho, RO Brazil; 7 Programa de Pós-Graduação em Conservação e uso de Recursos Naturais – PPGReN, Fundação Universida-de Federal de Rondonia, Porto Velho, RO Brazil; 8 Laboratório de Pesquisa Translacional e Clínica, Centro de Pesquisa em Medicina Tropical, Porto Velho, RO Brazil

**Keywords:** Ecology, Genetics

## Abstract

Large-scale surveillance and informed vector control approaches are urgently needed to ensure that national malaria programs remain effective in reducing transmission and, ultimately, achieving malaria elimination targets. In South America, *Anopheles darlingi* is the primary malaria vector and is responsible for the majority of *Plasmodium* species transmission. However, little is known about the molecular markers associated with insecticide resistance in this species. In this study, we developed a low-cost, high throughput amplicon sequencing (“amp-seq”) panel, consisting of 11 amplicons targeting genes linked to mosquito species identification (*cox-1* and *its2*) and insecticide resistance (*ace-1*, *GSTe2*, *vgsc* and *rdl*). When used in tandem with dual-index barcoding of amplicons, this approach permits high numbers of loci and samples to be sequenced in single runs, thereby decreasing costs and increasing efficiency. By screening 200 *An. darlingi* mosquitoes collected in Brazil, our amp-seq approach identified 10 point mutations leading to amino acid changes in *ace-1* (V243I, N294H, S673N, S674N/T) and *GSTe2* genes (I114V, D128E, T166I, T179I, and T205A). Overall, our work has demonstrated the utility of amp-seq to provide insights into the genetic diversity of *An. darlingi* mosquitoes. The amp-seq approach can be applied as a wide-scale insecticide-resistance surveillance technique to better inform vector-control methods.

**Supplementary Information:**

The online version contains supplementary material available at 10.1038/s41598-024-84432-x.

## Background

Malaria, caused by *Plasmodium* parasites and transmitted by *Anopheles* mosquitoes, is an important public health problem in the Americas, where ~ 505,600 cases were diagnosed in 2023 alone^1^. Approximately 1 in 5 of those malaria cases occurred in Brazil^1^, where the number of cases rose by 3% between 2015 and 2020 (137,982 vs. 142,112)^2^. The transmission of *Plasmodium* species in Brazil predominantly occurs in the Amazon region^2,3^. *Plasmodium vivax* parasites cause the majority (~ 83%) of malaria cases, followed by *P. falciparum* (~ 17%), and then other *Plasmodium* species (0.1%). Over the last 7 years, the proportion of cases caused by *P. falciparum*, the species that causes most severe disease, has increased by 7%^2^.

The primary malaria vector in Brazil, *Anopheles darlingi* (also called *Nyssorhynchus darlingi*), is highly susceptible to *Plasmodium* infection and can maintain malaria transmission even when parasites are at low densities^4–9^. This mosquito species is highly adaptable to recently anthropized environments and exhibits both exophagic and endophagic behavior. *An. darlingi* primarily feeds on humans and can occupy ecological niches left empty by other *Anopheles* species (spp.)^8,10^. Since 2017, deforestation has increased in the Brazilian Amazon, with previous studies showing these newly deforested areas or “frontiers” have higher *An. darlingi* abundance and increased malaria transmission^11^. The adaptability of *An. darlingi* mosquitoes is believed to be leading to year-round malaria transmission. Previously, forest populations of *An. darlingi* would peak during, and towards the end of the rainy season due to the increased availability of larval habitats in flooded areas near rivers. However, environmental changes produced by humans have created permanently available larval habitats, thereby supporting perennial malaria transmission^12,13^. These challenges represent a clear risk for Brazil to accomplish its National Elimination plan and achieve the World Health Organization (WHO) goal of reducing malaria cases by 90% within the next 7 years^14^.

Typical malaria control practice in Brazil uses insecticides, especially indoor residual spraying (IRS) or long lasting insecticide treated nets (LLINs)^15,16^. Pyrethroids such as cyperpermethrin and deltamethrin, have been the insecticide class applied in recent years using both methodologies, but organophosphates (malathion) and carbamates are used on *Aedes aegypti* as part of arbovirus control. Since the reintroduction of dengue in Brazil and the occurrence of outbreaks of other arboviruses (e.g., Zika and chikungunya), *Ae. aegypti* has been part of a national insecticide resistance screening programme to optimise elimination strategies, but no such programme exists for *Anopheles*^17,18^. Resistance to pyrethroids has been reported across *Anopheles* species globally, and for *An. darlingi* in countries surrounding Brazil, including Bolivia, Peru, Colombia, and French Guiana^19–21^. Resistance to carbamates has also been reported in Peru and Bolivia, and organochloride resistance has been observed in Peru and Colombia^22,23^. No resistance has been reported for organophosphates in Brazil, and no data is available for pyrroles or neonicotinoids. The lack of reporting on insecticide resistance in *An. darlingi* is a cause for concern, due to the high levels of associated *Plasmodium* spp. transmission by this vector.

The main insecticide resistance mechanisms observed by mosquito species are target site, metabolic and cuticular, and behavioural avoidance^24^. Target site resistance is mediated by mutations in insecticide target genes, such as the acetylcholinesterase-1 (*ace-1*), γ-aminobutyric acid (GABA) receptor (*rdl*), and voltage-gated sodium channel (*vgsc*). Particularly well-studied are the knockdown resistance (*kdr*) mutations associated with Dichlorodiphenyltrichloroethane (DDT) and pyrethroid-based insecticides, including the L1014 mutation in *vgsc*^25,26^. Point mutations in the glutathione-s-transferase epsilon (*GSTe2*) gene, which encodes an insecticide metabolising enzyme, are also associated with these insecticides, and the L119F and I114T mutations lead to resistance to permethrin in *An. funestus*^27,28^. Mutations and duplications in the *ace-1* gene result in organophosphate and carbamate resistance, and amino acid alterations in *rdl* have been associated with resistance to organochlorines, particularly dieldrin^29–31^. Molecular surveillance studies of insecticide resistance mutations in *An. darlingi* are scarce, with only a few investigations exploring target regions in *vgsc* and *ace-1* genes^32,33^. None of the single nucleotide polymorphisms (SNPs) previously found to be associated with insecticide resistance in other *Anopheles* species have been observed in *An. darlingi*, including in pyrethroid resistant populations^33^. It is possible that genetic variants in other regions within these genes or in additional candidate genes, may contribute to the observed outcomes.

Whole genome sequencing (WGS) has been applied to many vectors to better understand their genomic landscapes and identify candidate genes to unravel mechanisms of insecticide resistance. However, *Anopheles* genomes are large (~ 300 Mbp), and WGS is an expensive method that requires high quantities of DNA to gain suitable genomic insights, meaning it is inappropriate as a high-throughput surveillance method. The application of next-generation sequencing to targeted PCR amplicons, in tandem with dual-index barcoding, has been successfully used in other *Anopheles* spp., *Aedes* spp., and *Plasmodium* spp., as a high-throughput and low-cost screening method for insecticide or drug resistance mutations in target *loci*^34–37^. Targeting several candidate genes in many samples permits the tracking of emerging resistance and spread of known mutations in the population. This approach also allows for an analysis of genotype-phenotype associations to identify novel mutations linked to insecticide phenotypic assays.

Here we have designed an amplicon-sequencing (“amp-seq”) assay, consisting of a panel of 11 amplicons (each ~ 500 bp) covering multiple regions across 4 genes (*vgsc*, *ace-1*, *rdl*, and *GSTe2*) commonly associated with insecticide resistance, and a further 2 genes (*Its2* and *cox1*) used for species identification and phylogenetic analysis. Population genetics studies of *An. darlingi* have focused on *cox1* and *its2*, therefore we include both markers to compare the genetic structure of the *An. darlingi* populations examined here with existing available data from other countries. This approach allows for a broader understanding of the species’ genetic diversity across regions.

This multitarget assay was used to screen 200 *An. darlingi* mosquitoes collected in Brazil, revealing several new mutations. Our assay represents a cost-effective method to confirm mosquito species and conduct insecticide resistance surveillance, with the potential to inform control strategies for an understudied vector that is responsible for high levels of malaria transmission in South America.

## Methods

### Sample collection, species identification and DNA extraction

*An. darlingi* field mosquitoes were mostly collected from localities in Rondônia state (n = 171; Candeias do Jamari n = 116 (GPS: 8°39’41.0” S 63°01’58.8” W, 8°41’00.5” S 63°11’08.8” W), Porto Velho (GPS: 8 47’08” S, 63 55’04” W) *n* = 55) but we also include four samples from the Amazonas state (*n* = 4; Manaus *n* = 1 (GPS: − 2°58’52.7"S 60°03’03.4"W), Manacapuru (GPS: 3°16’34.3"S 60°37’36.9"W) *n* = 3)^38,39^. The collections were performed during studies of vector density in malaria endemic areas of Rondônia 2018–2019^38,39^. Mosquitoes from the *An. darlingi* colony of Porto Velho/Rondônia (colony generations : F2-F4, F9-F11, F21, F33-F35, F39, F40-F42) were also included in the study (*n* = 25)^40^. In total, 200 mosquitoes were screened. The field samples were selected based on whether the localities had used insecticides (Porto Velho/ Manaus) or had little/no recent insecticide usage (Candeias do Jamari, Manacapuru). During the sample collection period in Porto Velho, alpha-cypermethrin was applied for both IRS and LLINs, while cypermethrin was used for thermal fogging. In Manaus, etofenprox was employed for IRS, and alpha-cypermethrin was applied to LLINs. For thermal fogging, both cypermethrin and lambda-cyhalothrin were used. The specimens were initially identified by stereoscopic microscopy, using the established dichotomous keys^41^. Genomic DNA was extracted from whole mosquitoes using the Qiagen DNeasy ^®^ Tissue and Blood kit (Qiagen, Hilden, Germany), according to the manufacturer’s instructions. Briefly, each mosquito was grounded with 30 µL of lysis buffer and the DNA extract was resuspended in 50 µL elution buffer.

### Primer design

Amplicon primers were designed using Primer3 software, against sequences downloaded from VectorBase^42^. The primers aimed to amplify an approximate 500 bp region containing SNPs previously described as associated with insecticide resistance in *Anopheles* or *Aedes* mosquito’s species. This resulted in a panel of 9 primers targeting 4 genes: (i) *vgsc* (4 amplicons, targeting four domains); (ii) *rdl* (2 amplicons, targeting 3 SNPs); (iii) *ace-1* (2 amplicons, targeting two SNPs, including G280S^32^); and (iv) *GSTe2* (1 amplicon, targeting two SNPs). When possible, these primers were designed to bind to exonic regions. Two other amplicons were designed to target genes commonly used for species identification and phylogenetic investigation: the ribosomally encoded gene internal transcribed spacer 2 (*its2*); and cytochrome c oxidase I (*cox1*), a locus found in the mitochondria. This resulted in a final panel of 11 amplicons, covering 6 genes (Table 1). Each primer sequence was concatenated with a unique 5’ barcode (8 bp) designed in house^34^. Samples were assigned a unique forward and reverse barcode combination used for the generation of amplicons to enable sample pooling before sequencing. To identify amplicons suitable for multiplexing the ThermoFisher Scientific Multiple Primer Analyser was used with sensitivity for dimer detection set to one.

**Table 1:** Primers and targets of *An. darlingi* amp-seq panel.

*Anopheles darlingi* Primers								
Aim	Target Gene	Amplicon	Accession ID	Target SNP*	Exon Span	Forward primer	Reverse Primer	Product Size (bp)
**Insecticide** **Resistance**	*vgsc*	VGSCI	ADAC011160	V416L	9–10	GCCTTTCGTCTAATGACTCAAGA	GCCAAGATTAAATTTACAAGGTAAAAC	500
		VGSCII		L1014F	20–21	ACCGTTTCCCCGATAAAGAC	ACGGACGCAATTTGACTTGT	450
		VGSCIII		F1511C/ N1552Y	30	TTTTCCAGGTTGCCACTTTC	ATTGCTTGTGGCCTCCACT	475
		VGSCIV		D1739Y	32–33	AAAATATTTCGTTTCCCCAACA	TCCCAGGATAACCTTTGTCG	447
	*ace-1*	ACE1_1	ADAC000377	G305S	2	TAAGAAGGTGGACGTGTGGC	AGAGCAAGGTTCTGATCGAA	450
		ACE1_II		N642I	4–5	GACGGGGTACGTCGACAA	AAGGCGCTACTTTCACACG	500
	GSTe2	GSTe2	ADAC008205	L119F	3	TTCGAATCCGGTGTGATCTT	TGGTCACGATCATCTTTATTGG	471
	*rdl*	RDL1	ADAC005672	A296S/ V371I	7	CACCAACACCAGTCTGATCG	TGGCAAATACCATGACGAAG	490
		RDL2		T345S	8	TGGTTTTTCCCAATCGTTTT	CTGCCCATCTGCTGCTTC	492
**Phylogeny**	*cox-*1	COX-1	HM022406.1	n/a	n/a	TCTCCAGGGATTACTTTAGATCG	GCTGGGCTGTATGTTAATTGAG	494
	ITS2	ITS2	KF436940.1	n/a	n/a	GACTCAGTGCGAGGTACACA	GAGGCCCACTTGAGATCCTA	455

*Target SNP loci in *An. darlingi*.

### Amplicon generation

Multiplex Polymerase chain reactions (PCR) were performed using NEB Q5 hot start polymerase (New England BioLabs, UK) with a total volume of 25µl per reactions. Sample volume of 1µL (~ 2ng/µL) was used, with an average final primer concentration of 0.5µm in each PCR. The amplification was conducted as follows: hot-start polymerase activation for 3 minutes at 95 °C, followed by 30 cycles of 95 °C for 10 seconds, 58 °C for 30 seconds and 72 °C for 45 seconds, followed by a final elongation step of 72 °C for 5 minutes. Post-multiplex PCR reaction, amplicons were visualised on a 1% agarose gel to confirm amplification, alongside band size and intensity. Amplicons for each sample contained a unique 5’ barcode (8 bp) designed in-house^34^, making it possible to pool all amplicons across samples. Pools were purified using Roche Kapa beads following manufacturer’s instructions. A bead to sample ratio of 0.7:1 was used to remove excess primers and PCR reagents. The Qubit 2.0 fluorimeter HS DNA kit was used to quantify the pool concentration. Illumina barcodes and adaptors were further added to the sample pools as part of the Illumina-based Amplicon-EZ service (Genewiz, UK). Pools contained a maximum of 200 amplicons to maximise coverage. Each indexed pool was sequenced using a 2 × 250 bp (paired-end) configuration on an Illumina MiSeq. A minimum of 50,000 paired-end reads were attained per pool, which equates to at least 450 reads per amplicon in a pool of 110 amplicons, at a cost of < US$0.5 per amplicon. For variant confirmation with Sanger sequencing, amplicons were generated using the same primers but were amplified in simplex reactions, before being sequenced by Genewiz, UK.

### Amplicon analysis

Raw fastq files were de-multiplexed using the unique barcode combination assigned to each pool and each sample, using an in-house python script (https://github.com/LSHTMPathogenSeqLab/amplicon-seq/blob/main/scripts/amplicon_script.py). The resulting sample fastq files were then analyfirst trimmed using Trimmomatic software, then mapped to the reference sequence (idAnoDarlMG_H_01, from NCBI: https://www.ncbi.nlm.nih.gov/datasets/genome/GCF_943734745.1/) using the bwa-mem package, and reads are then filtered using Samclip software^43–45^. GATK HaplotypeCaller (v4.1.4.1, default parameters) and Freebayes (v1.3.5, --haplotype-length − 1) software were used to call variants^46,47^. The SNPs and small insertions/deletions (INDELs) identified were then filtered using bcftools^48^. To pass quality control checks, a minimum depth of 30 reads, phred score of > 30 per base, and a minimum allele depth of 10 was required. Variants had to be present in > 1 sample, and across > 1 of the sample pools sequenced. The SnpEff tool was applied to annotate variants using a database built from the idAnoDarlMG_H_01 reference genome^49^. Variants were then genotyped based on the proportion of alternative allele to total depth coverage, called as homozygous reference (< 20% alternate allele reads), heterozygous (20–80% alternate allele), or homozygous alternate (> 80% alternate allele reads)^34^.

### Phylogenetic analysis

For the *Its2* and *cox1* amplicons, SNP calls with > 50-fold read depth were converted to fasta files using an in-house pipeline (https://github.com/LSHTMPathogenSeqLab/fastq2matrix). Only sequences with SNP calls that reached this depth were included in phylogenetic analysis. For each gene, sequence data was aligned using the MAFFT tool. Sequences from the NCBI from other countries were included in the resulting alignments^50^. For *cox-1*, 67 sequences were added from Brazil, Honduras, Belize, Colombia, Panama, Ecuador, and Peru. For *its2*, an additional 26 sequences were aligned from Brazil, Colombia, Belize, and Bolivia. For tree generation, sequences from both genes were concatenated, and the resulting alignments were viewed and trimmed using Aliview^51^. Phylogenetic trees were constructed using RAxML software^52^. The trees were built using a maximum-likelihood method, with the GTRGAMMA option. This approach assumes a GTR model of nucleotide substitution, and a gamma model of rate heterogeneity. A bootstrap value of 1000 was used for tree construction, and the resulting tree was visualised using iTOL software^53^.

### Haplotype networks and maps

To construct the haplotype network, fasta sequences for *cox-1* and *its2* were aligned for all samples, and then the packages Pegas and ggplot2 R^54,55^ were applied. Pegas was used to calculate nucleotide diversity, haplotype diversity, Tajima’s D statistic, fixation indel (Fst), heterozygosity and linkage disequilibrium^55^.

## Results

### Detection of novel SNPs in genes associated with insecticide resistance

A total of 200 *An. darlingi* samples were sequenced, with the resulting average amplicon coverage ranging from 171- to 5621-fold (Table 2). From the alignments, 246 SNPs and 20 INDELs passed all quality control measures, the majority of which were either synonymous (37.8%) or intronic variants (45.9%). Ten SNPs and one INDEL were annotated as non-synonymous and resulted in an amino acid change (Table 3 & Supplementary Table S1). Missense SNPs were only found in *ace-1* and *GSTe2* genes and all were present in at least two samples and in two or more populations (Table 3). These SNPs have not been previously reported. In the *ace-1* gene, 111 SNPs were found across the two amplicons, including five missense SNPs (V243I, N294H, S673N, S674N, and S674T), with S674N occurring at the highest frequency (19.6% heterozygous and 2.7% homozygous samples). Three of these five mutations (V243I, N294H and S674T) occurred only in field populations, while S673N and S674N appeared in field populations and all colony samples (Table 4). A further five non-synonymous SNPs were found in the *GSTe2* gene. Three of the missense SNPs (I114V, T166I, and T179I) occurred at frequencies below 23%, and two others (D128E and T205A) appear to be at or approaching fixation as no samples were genotyped as homozygous reference, and over 90% were homozygous alternate for both amino acid substitutions. Two of these amino acid alterations in the *GSTe2* gene were found only in field populations (I114V and T166I), and the remaining three (D128E, T191I, and T205A) were also observed in colony samples.

**Table 2:** Average amplicon coverage, and number of genetic variants identified.

Amplicon	Average coverage	Number of SNPs	Number of Non-synonymous SNPs	Number of INDELs	Number of Non-synonymous INDELs
ACE1_I	3190.40	43	2	0	0
ACE1_II	1415.81	68	3	12	0
COI	1996.52	20	0	0	0
GSTe2	3196.05	40	5	1	0
ITS2	5621.32	6	0	0	0
VGSCI	272.17	9	0	3	1
VGSCII	216.81	4	0	0	0
VGSCIII	780.34	4	0	0	0
VGSCIV	1620.89	1	0	2	0
Rdl1	171.17	16	0	1	0
Rdl2	895.84	35	0	1	0

**Table 3:** Locations and allelic frequencies of detected non-synonymous variants.

Chromosome	Amplicon	Position	Sample number	Annotation	Genotype frequencies			Allele frequencies	
					0/0	0/1	1/1	0	1
**SNPs**									
NC_064874.1	ACE1_I	15,679,573	191	Val243Ile	92.67	6.81	0.52	96.07	3.93
		15,679,726	191	Asn294His	97.38	2.62	0	98.70	1.30
	ACE1_II	15,681,121	149	Ser673Asn	95.30	4.03	0.67	97.32	2.68
		15,681,124	148	Ser674Asn	77.70	19.60	2.70	87.50	12.50
		15,681,124	145	Ser674Thr	98.64	0.68	0.68	98.97	1.03
	GSTe2	89,825,807	129	Ile114Val	95.35	4.65	0	97.67	2.33
		89,825,922	129	Asp128Glu	0	7.75	92.25	3.88	96.12
		89,826,035	128	Thr166Ile	87.50	12.50	0	93.75	6.25
		89,826,074	128	Thr179Ile	78.91	19.53	1.56	88.67	11.33
		89,826,151	129	Thr205Ala	0	6.20	93.80	3.10	96.90
**INDELs**									
NC_064875.1	VGSCI	35,317,107	40	Ile422del	67.5	30.0	2.5	82.5	17.5

**Table 4:** Genotype frequencies of non-synonymous SNPs in the four field collection sites.

Amplicon	SNP position	Amino acid change	State of Rondonia						State of Amazonas					
			Porto Velho* (50)			Candeias do Jamari (*n* = 113)			Manacapuru (*n* = 3)			Manaus* (*n* = 1)		
			0/0	0/1	1/1	0/0	0/1	1/1	0/0	0/1	1/1	0/0	0/1	1/1
**ACE1_I**	**15,679,573**	**Val243Ile**	90.0%	8.0%	2.0%	93.8%	6.2%	0.0%	66.7%	33.3%	0.0%	0.0%	100%	0.0%
	**15,679,726**	**Asn294His**	96.0%	4.0%	0.0%	97.3%	2.7%	0.0%	100%	0.0%	0.0%	100%	0.0%	0.0%
**ACE1_II**	**15,681,121**	**Ser673Asn**	100%	0.0%	0.0%	96.4%	2.4%	1.20%	66.7%	33.3%	0.0%	0.0%	100%	0.0%
	**15,681,124**	**Ser674Asn**	78.3%	17.4%	4.3%	77.4%	20.2%	2.40%	100%	0.0%	0.0%	100%	0.0%	0.0%
	**15,681,124**	**Ser674Thr**	97.7%	0.0%	2.3%	98.8%	1.2%	0.0%	100%	0.0%	0.0%	100%	0.0%	0.0%
**GSTe2**	**89,825,807**	**Ile114Val**	97.6%	2.4%	0.0%	93.3%	6.7%	0.0%	100%	0.0%	0.0%	100%	0.0%	0.0%
	**89,825,922**	**Asp128Glu**	0.0%	4.9%	95.1%	0.0%	10.7%	89.3%	0.0%	0.0%	100%	0.0%	0.0%	100.0%
	**89,826,035**	**Thr166Ile**	90.2%	9.8%	0.0%	86.5%	13.5%	0.0%	66.7%	33.3%	0.0%	100%	0.0%	0.0%
	**89,826,074**	**Thr179Ile**	80.5%	17.1%	2.4%	77.0%	23.0%	0.0%	66.7%	0.0%	33.7%	100%	0.0%	0.0%
	**89,826,151**	**Thr205Ala**	0.0%	4.8%	95.2%	0.0%	8.0%	92.0%	0.0%	0.0%	100%	0.0%	0.0%	100%

*indicates insecticides are regularly used in this locality.

For v*gsc*, the analysis revealed a INDEL caused by a 3 bp deletion, resulting in an isoleucine deletion at position 422 in the first domain. This mutation has not previously been reported and occurred at a frequency of 25% across field and colony samples. Sanger sequencing confirmed these mutations, ruling out sequencing artifacts. No other non-synonymous variations were observed across the four amplicons investigated in the *vgsc* gene. No missense polymorphisms were detected in the *gaba* gene (*rdl*).

### Genetic diversity of *An. darlingi* populations in Brazil

Sequences for mitochondrial *cox-1* gene and ribosomal *its2* were generated for genetic diversity analysis. In the *its2* gene, six SNPs were identified. Originally a total of 91 SNPs were identified in the *cox-1* amplicon, but upon further inspection, 71 of these SNPs were present in one sample (AnDar600), which was subsequently identified as an *An. peryassui* isolate (Blast score: 99.2% identity) and excluded from further analysis. Twenty SNPs were identified in the *cox-1* gene, 19 of which appeared in the Candeias do Jamari population, 13 in colony samples, 10 in the Porto Velho populations, and six in the State of Amazonas samples. A fixation index analysis for each SNP revealed no significant population differentiation across these genes (Fst < 0.032).

**Fig. 1:**
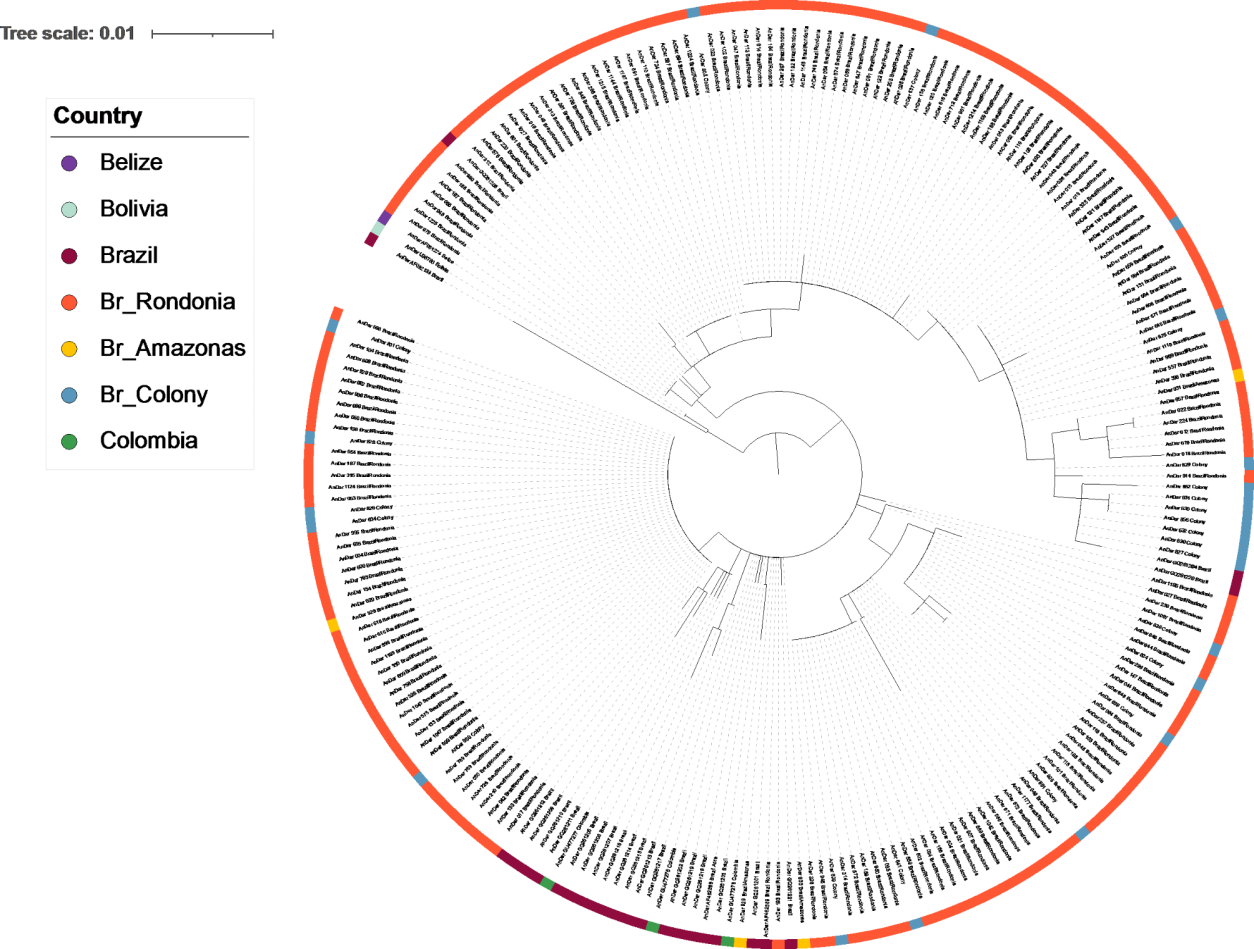
Maximum-likelihood tree constructed using *its2* gene sequences generated in this study (total = 198, Br_Rondonia = 171, Br_Amazonas = 4, Br_Colony = 23), alongside other publicly available *An. darlingi its2* sequences (*n* = 26, Brazil, Colombia, Belize, and Bolivia). The tree was built using the maximum-likelihood method assuming GTR model of nucleotide substitution, with the gamma model of heterogeneity rate.

Phylogenetic analysis with the *its2* gene reflected the small number of SNPs shown in these populations, and very little differentiation was observed between Brazilian isolates and those from other countries (Fig. 1). The tree separated into two main clades, the first of which contains three publicly available samples from Brazil, Belize, and Bolivia. The second clade contained several subclades that included all sequences generated in this study (*n* = 198) along with the remaining publicly available Brazilian and Colombian samples (*n* = 26). No differentiation between the different Brazilian populations was observed, which was supported by low nucleotide diversity (*π* = 0.00536) (Table 5).

**Table 5:** Nucleotide and haplotype diversities of ITS2 per population.

Population	Nucleotide diversity	Haplotype diversity
**All (n = 198)**	0.00536	0.893
**Amazonas (n = 4)**	N/A	N/A
**Rondônia (n = 172)**	0.00607	0.883
**Colony (n = 23)**	0.00639	0.913

**Fig. 2:**
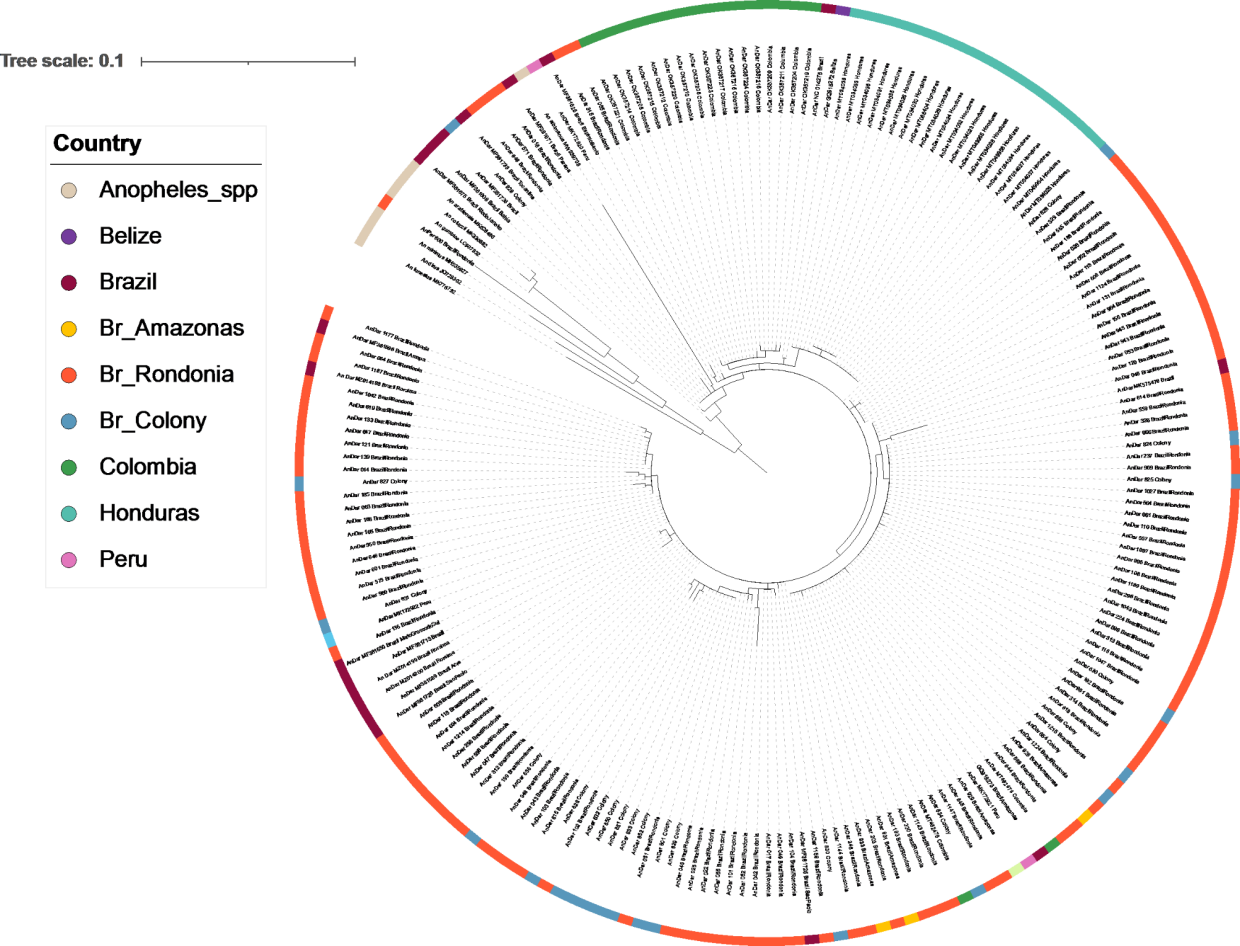
Maximum-likelihood tree constructed using *cox-1* gene sequences generated in this study (total = 129, Br_Rondonia = 106, Br_Amazonas = 3, Br_Colony = 20), alongside other publicly available *An. darlingi cox-1* sequences (*n* = 67, Brazil, Honduras, Belize, Colombia, Panama, Ecuador, and Peru). This tree also has a group of *Anopheles* spp. including *An. albimanus*, *An. arabiensis*, *An. coluzzi*, *An. dirus*, *An. funestus*, and *An. gambiae.* The tree was built using the maximum-likelihood method assuming GTR model of nucleotide substitution, with the gamma model of heterogeneity rate.

For the *cox-1* gene, a total of 128 sequences from this study alongside 67 publicly available samples were analised. (Fig. 2). Using this gene, it was possible to see clusters separating most Colombian, Honduran and Brazilian samples. Although, sequences from other countries can be seen interspersed particularly within the Brazilian *cox-1* sequences. There was both low genetic and nucleotide diversity (0.00697) in *cox-1* (Table 6), similar to the *its2* results.

**Table 6:** Nucleotide and haplotype diversities of *cox-1* per population.

Population	Nucleotide diversity	Haplotype diversity
**All (n = 129)**	0.00653	0.856
**Amazonas (n = 4)**	N/A	N/A
**Rondônia (n = 105)**	0.00688	0.867
**Colony (n = 20)**	0.00611	0.837

A phylogenetic based on concatenated *its2* and *cox-1* sequences revealed little differentiation between samples across the geographical regions (Supplementary figure S1). Both loci demonstrated high haplotype diversity (Tables 5 and 6), with 44 haplotypes identified for the c*ox-1* gene, and 31 for *its2*. Most of the haplotypes, 77.3% for *cox-1* (*n* = 34), and 54.8% for *Its2* (*n* = 17), were present in only one sample (singletons). The high number of singleton haplotypes reflects the high proportion of SNPs occurring at low frequency in the populations. Haplotype 34, representing the *cox-1* gene, was the most frequent, and was present in samples from the colony, and states of Amazonas and Rondônia (Supplementary Figure S2). A higher number of *its2* haplotypes (*n* = 14, 45.2%) were present in more than one sample, compared to *cox1* (*n* = 10, 22.7%). Haplotypes 1 and 9 were the most frequent, present in both colony and state of Rondônia populations (Supplementary Figure S3). The samples from the state of Amazonas shared haplotypes with both colony and state of Rondônia samples. The networks revealed shared haplotypes for both genes across the three populations, and included several other samples available from other Brazilian states.

## Discussion

The application of our amplicon sequencing panel to Brazilian field and colony *An. darlingi* samples has demonstrated its potential utility for species identification, and the discovery of SNPs in genes associated with insecticide resistance. Whilst no previously reported SNPs associated with insecticide resistance in other *Anopheles* species were found in this study, ten other non-synonymous SNPs were detected. Of the five SNPs found in the *GSTe2* gene, all except one (D128E) are either in amino acid positions that are highly variable across *Anopheles* spp. or the mutation results in a change to an amino acid that is present in the reference of another *Anopheles* spp. The I114V substitution is in the same location as a previously reported amino acid alteration in *An. gambiae*^56^. In *An. gambiae*, the mutation results in a I114T substitution, which is hypothesised to cause resistance through the introduction of hydroxyl (-OH) group on the substituted threonine. The hydroxyl group decreases product affinity in the hydrophobic DDT binding site, thereby increasing metabolic turnover of the insecticide. In this instance the valine substitution does not introduce this same hydroxyl group, and valine is present as a reference amino acid in *An. atroparvus.* The D128E mutation occurs at a highly conserved site across *Anopheles* spp., with aspartic acid (D) present as the reference for all. The alteration to glutamic acid results in a similar amino acid structure with the addition of an extra carbon. This mutation appears to be near fixation as 92% of samples were genotyped as homozygous alternate, and the remaining 8% as heterozygous.

Pyrethroids are the predominant insecticide class being applied for malaria-focussed vector control in Brazil^16^. This implies that if target site mutations were to arise, they would predominantly occur in the *vgsc* gene. However, in Brazilian dengue control programmes, *Aedes* spp. are targeted with both pyrethroids and organophosphates, and this usage could impact *Anopheles* spp. While malaria and Aedes-transmitted arboviruses tend to occur in different ecological zones in Brazil—malaria is more prevalent in rural areas while arboviruses are more common in urban environments—there can still be some overlap in the intervention areas. This overlap is especially evident in peri-urban regions and towns where both vector species might be present and where rapid urbanization and migration could increase the risk of both diseases. This could explain the three non-synonymous SNPs found in the *ace-1* gene, a target for organophosphates. The previous reported target-site mutations G119S and N485I (positions in *Torpedo califonica*, G305S and S642I) alter susceptibility to the organophosphate and carbamate classes of insecticide^29^. *An. darlingi* resistant to carbamates have been reported in Bolivia, close to the Brazilian border^22^. Of the five amino acid alterations detected in the *ace-1* gene (V243I, N294H, S673N, and S674N/T), in the *Anopheles darlingi* from our study, none have been previously reported. The S673N and S674N/T *ace-1* mutations occur approximately 49 residues away from one of the three catalytic sites (H440; H625 in *An. darlingi*) and were present in field and colony samples for which the resistance status is not known. The N294H alteration occurs 9 amino acids upstream of the G119S (G305S) mutation, and results in a change from an amino acid with a polar uncharged side chain (asparagine) to one with a charged side chain with an aromatic imidazole ring. For all five of these amino acid substitutions, the reference amino acid is conserved across other *Anopheles* species (results from sequence alignment of *An. darlingi*, *An. albamanus*, *An. gambiae*, *An. funestus*, *An. stephensi*, and *An. minimus*). An amino acid conserved across species suggests is could be important for protein function. Further studies, which include bioassays, are needed to confirm if these mutations result in organophosphate or carbamate resistance. It is not possible at present to exclude potential candidate SNPs on the basis of their presence in the colony samples, as their insecticide resistance profiles are still under evaluation.

The four study locations had varying insecticide usage, with two sites, Porto Velho (State of Rondônia), and Manaus (State of Amazonia), having a history of intensive use of pyrethroids and carbamates, particularly the application of cypermethrin related counpounds for residual spraying effors, with lambda-cyhalothrin used since 2012. In contrast, Candeias do Jamari (State of Rondônia) and Manacapuru (State of Amazonas), had little to no insecticide spraying. All missense SNPs identified in this study were found in locations with intensive insecticide spraying, and locations with little to no insecticide usage. Additional studies that combine phenotypic and molecular surveillance data are needed to understand the impact of the SNPs detected here on insecticide resistance in *An. darlingi.*

The lack of SNPs found in the *rdl* gene may reflect the reduced selective pressure exerted by dieldrin.

Mutations in *gaba* result in dieldrin resistance, an organochloride that has been prohibited from use for at least the past decade due to its adverse effects on human health. However, *rdl* SNPs have been documented in other *Anopheles* species, which also have no longer exposure to dieldrin. These mutations highlight the potential for persistence in populations even after the cessation of insecticide use, driven by environmental residues or cross-resistance with other insecticides. Continued monitoring of *rdl* SNPs across *Anopheles* species is essential to understand the dynamics of resistance and its potential implications for vector control strategies. In relation to the *vgsc* gene, four amplicons were investigated, but no *kdr* mutations were detected in the populations surveyed here. This observation was also reported in pyrethroid resistant populations of *An. darlingi* from Colombia^33^. The lack of *kdr* mutations suggests that SNPs in v*gsc* may play a reduced role in *An. darlingi* insecticide resistance compared to other *Anopheles spp.*, such as *An. gambiae*. An INDEL resulting in isoleucine deletion at position 422 was detected in 25% of sample, across both field and colony sources. Further studies are needed to understand the involvement of this deletion in insecticide resistance.

The lack of known resistance associated SNPs in *An. darlingi* could be due to the gene flow between geographically close populations with varying levels of insecticide use. The mixing of possible resistant populations with insecticide-sensitive populations may minimise the frequency of resistant alleles, a dilution effect hypotesised by Vezenegho et al.^19^. Another hypothesis is that other genetic variants may play a role in resistance, or that different mechanisms, such as differential gene expression, could be involved in this species resistance status.

In relation to the genetic diversity across populations, there was little differentiation between *An. darlingi* from Brazil and other regions using *its2* gene data. However, the phylogenetic tree constructed using *cox-1* gene data revealed that samples generally grouped by country. It has been hypothesised that physical barriers like the Atlantic forest mountain range or the Amazon River, prevent the mixing of these populations and so they appear genetically distinct^57–59^. Data from the *cox-1* gene revealed an outlying sample, which aligned with another Anopheleles species (*An. peryassui*).

Within the Brazilian population, low nucleotide diversity was seen in tandem with high haplotype diversity for both *cox-1* and *its2* genes, indicating many low frequency variants. This observation is consistent with previous studies conducted on *cox-1* across Central and South American, and within Brazilian and Colombian *An. darlingi* populations^13,57,60^. The geographical proximity of the collection sites of these mosquitoes may also contribute to the low genetic diversity observed here. The inclusion of study sites that are more geographically distant or from other countries may give greater resolution to the population dynamics of this species.

Overall, our amp-seq panel provides a tool to investigate the genetic diversity of this understudied *An. darlingi* vector. It is a high-throughput, low-cost assay for species identification and the detection of novel SNPs in insecticide resistance associated genes. Further investigation is required to identify whether these SNPs contribute to insecticide resistance in *An. darlingi*. A clear limitation of this method is that it currently only includes target regions of known loci associated with insecticide resistance. However the panel is easily adaptable, enabling the inclusion of additional targets in these or other genes, including further metabolic markers. The panel can be used in tandem with phenotypic assays to identify SNPs that result in functional changes. Large-scale surveillance methods are urgently needed to inform malaria vector control methods in Brazil, particularly to assist initiatives to reduce malaria transmission. Our panel represents the first steps towards a high-throughput, multitarget molecular surveillance method for tracking known and identify potential new markers of resistance.

## Supplementary Information

Below is the link to the electronic supplementary material.

Supplementary Material 1

## Data Availability

All raw sequence data is listed in the European Nucleotide Archive (Project ID: PRJEB61194, Accession numbers: ERR11204754 to ERR11204953).
