# Supplementary material for: Application of a targeted amplicon sequencing panel to screen for insecticide resistance mutations in Anopheles darlingi populations from Brazil
Source: Sci Rep. 2025 Jan 3;15:731. doi: 10.1038/s41598-024-84432-x (PMC11698964; doi:10.1038/s41598-024-84432-x)
Supplement: Supplementary file 1 — Supplementary Material 1 [file 41598_2024_84432_MOESM1_ESM.docx]

**Supplementary figure S1.** Maximum-likelihood tree constructed using concatenated *its2* and *cox-1* gene sequences generated in this study (n=127). This included samples from Rondonia (n = 106), Colony (n=18), Amazonas (n=3). The tree was built using the maximum-likelihood method assuming GTR model of nucleotide substitution, with the gamma model of heterogeneity rate.

**
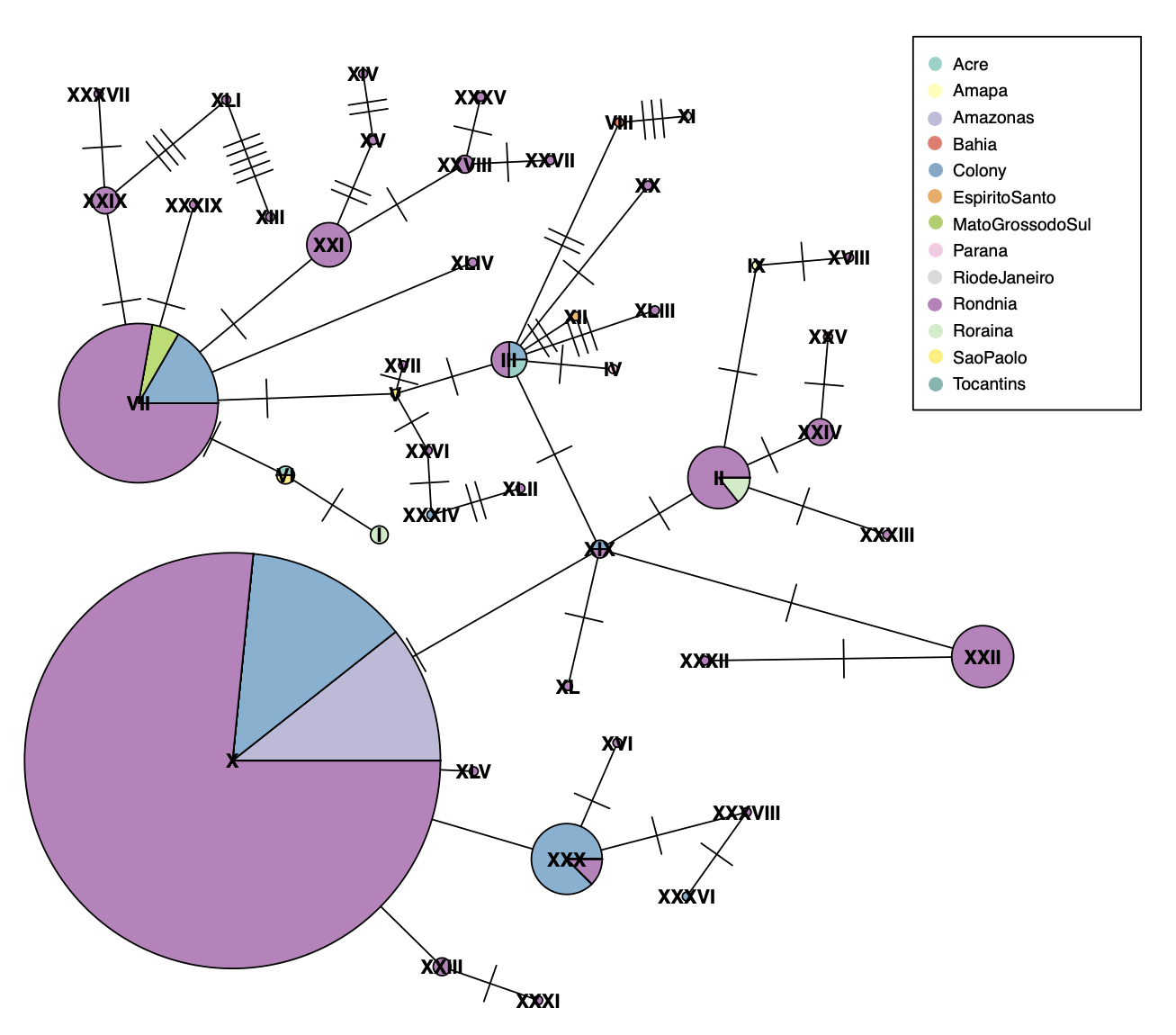
**

**Supplementary Figure S2.** Haplotype or minimal-spanning network constructed using *cox-1* sequences generated in this study and public available data from Brazil. Each node represents a haplotype, each segment within the node represents a region, and is proportionally sized to the number of sequences present in the segment and node. The number of number of ticks between nodes represents the number of genetic differences between nodes.

**
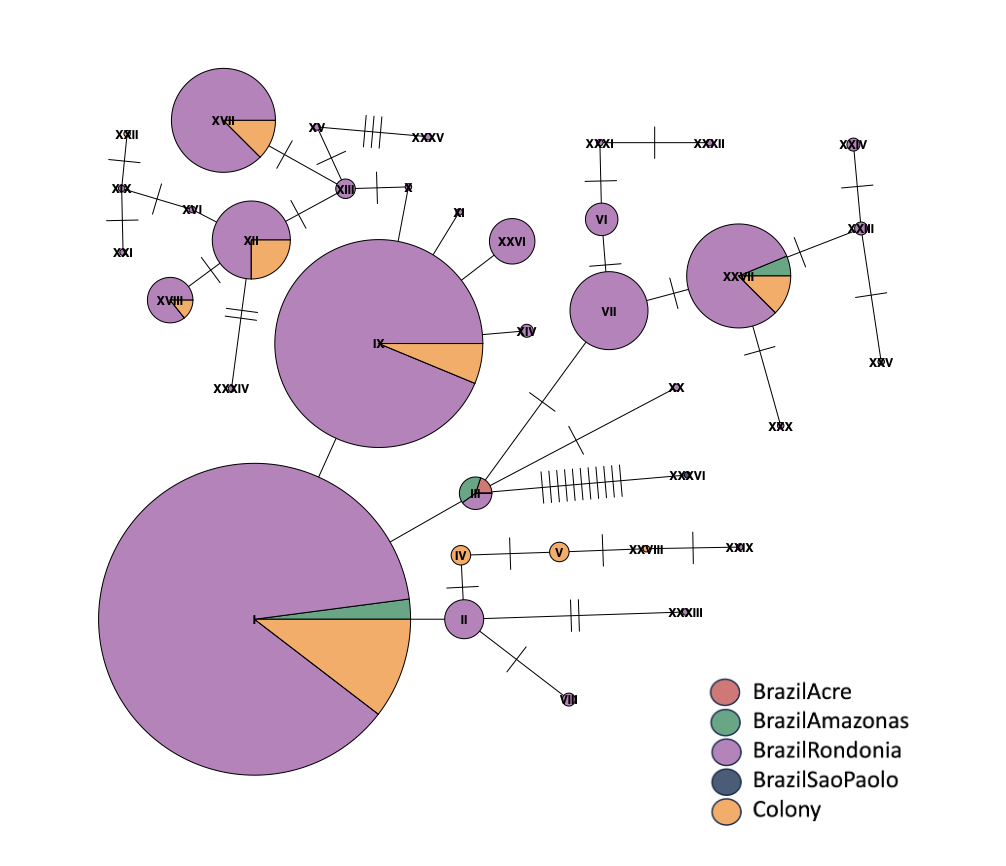
**

**Supplementary Figure S3.** Haplotype or minimal-spanning network constructed using its2 sequences generated in this study. Each node represents a haplotype, each segment within the node represents a region, and is proportionally sized to the number of sequences present in the segment and node. The number of number of ticks between nodes represents the number of genetic differences between nodes.

**Supplementary Table S1.** Number of genetic variants identified per population

| **Population** | **Sample Number** | **Number of SNPs** | **Number of Non-synonymous SNPs** | **Number of INDELs** | **Number of Non-synonymous INDELS** |
| --- | --- | --- | --- | --- | --- |
| All | 200 | 246 | 10 | 20 | 1 |
| Amazonas State | 4 | 91 | 7 | 14 | 0 |
| Rondônia State | 171 | 241 | 10 | 19 | 1 |
| Colony | 25 | 133 | 6 | 16 | 1 |
